# Auditory N1 Suppression and Omission N1 Do Not Share a Common Underlying Mechanism

**DOI:** 10.1111/psyp.70094

**Published:** 2025-06-19

**Authors:** Valentina Tast, Erich Schröger, Andreas Widmann

**Affiliations:** ^1^ Wilhelm Wundt Institute for Psychology Leipzig University Leipzig Germany

**Keywords:** auditory, EEG, N1 suppression, omission N1, predictive coding, sensory suppression

## Abstract

Recent theories describe perception as an inferential process based on internal predictive models that are adjusted by prediction violations (prediction error). Two modulations of the auditory N1 event‐related brain potential component have been interpreted as reduced or enhanced prediction error for predictable sensory input: The sound‐related N1 component is attenuated for self‐generated sounds compared to the N1 elicited by externally generated sounds (N1 suppression). An omission‐related component in the N1 time‐range is elicited when the self‐generated sounds are occasionally omitted (omission N1). We wanted to confirm that both N1 suppression and omission N1 are sensitive to the predictability of sound identity, as reported in the literature. We manipulated the predictability of sound identity in a self‐generation paradigm in which button presses in one condition always produced the same sound or in another condition produced a sound randomly selected from a large set of sounds. Omission N1 was modulated by manipulating the predictability of sound identity but surprisingly N1 suppression was not. This contradicts previous reports, challenges prediction‐related interpretations of the N1 suppression, and supports alternative explanations for N1 suppression like action‐related unspecific sensory gating.

## Abstract 2

Our study reports evidence that the auditory omission N1 reflects predictive processing. In contrast, our findings also provide evidence against a prediction‐related interpretation of the N1 suppression in the context of self‐generated sounds. Rather, it suggests that N1 suppression reflects unspecific sensory gating effects. Our results imply that omission N1 and N1 suppression are based on different mechanisms.

**Abbreviations**
EEG: electroencephalogram
EOG: electrooculogram
ERP: event related potential
fMRI: functional magnetic resonance imaging
IC: independent component
ICA: independent component analysis
MMN: mismatch negativity
oN1: omission N1
oN2: omission N2
oP3: omission P3
PCA: principal component analysis
PE: prediction error
ROI: region of interest
SOA: sound onset asynchrony

## 1 Introduction

Sounds which are generated by the perceiver are processed differently than sounds that happen to appear without the perceiver's intention. In many studies, this differential processing expresses in an alteration of brain activity for self‐generated sounds, for example, in a suppression of the auditory N1 event‐related potential (ERP) Component. The N1 suppression effect, first described by Schafer and Marcus (1973), manifests in a reduced ERP amplitude for the self‐produced sounds compared with externally produced sounds in the N1. This is supposedly a result of predictability: the self‐produced sound is more expected. Another phenomenon that relies on action‐effect predictability is the omission N1 that occurs in the unexpected absence of an expected sound (SanMiguel, Widmann, et al. 2013; Stekelenburg and Vroomen 2015). In the present study, we investigate if N1 suppression and omission N1 are sensitive to the predictability of the identity of the forthcoming sound.

Sensory suppression was usually explained by means of prediction related to auditory reafference (Crapse and Sommer 2008; Korka et al. 2022; Miall and Wolpert 1996). This interpretation is supported by findings showing a smaller N1 suppression when the pitch of the sound is unpredictable (Bäß et al. 2008; Knolle et al. 2013) and by a study on mice that showed strong suppressed neural responses only in response to tones that the mice learned to associate with an action and not to deviant tones (Audette and Schneider 2023).

In a broader context the N1 suppression (and related phenomena) is explained by theories describing perception as the result of an inferential process based on internal predictive models (Friston 2005; Rao and Ballard 1999), for example, the Predictive Coding Theory (Clark 2013; Friston 2005). The core idea is that the bottom‐up sensorial input is compared with the top‐down prediction, and this comparison is used to update inferences about the likely cause of this input in the external world. The difference between these two pieces of information is expressed as the prediction error (PE), which corresponds to transient ERPs such as the auditory N1 (Friston 2005). The “predictive brain” tries to minimize PE (via iterative bottom‐up and top‐down loops along the processing hierarchy, including the updating of the model and/or acquiring additional sensorial information). According to the predictive processing theory, the N1 for self‐generated sounds is attenuated, because the listener (who generated the sound) knows about the forthcoming sound, resulting in a better prediction, and thus, in a smaller PE (i.e., N1).

However, there are also studies showing that the N1 suppression might instead be a result of motor activity. A study on mice revealed a general suppression of auditory sensory processing during motor activity (Schneider et al. 2018) which is supported by studies in humans showing that for the N1 to be suppressed it is not necessary to recognize oneself as the agent for causing the sound as long as the sound coincides with motor activity (Horváth et al. 2012; Timm et al. 2016).

Studies investigating the N1 suppression still use a variation of the paradigm described by Schafer and Marcus (1973). In an active task, participants were asked to press a button in a steady rhythm to produce sounds. These sounds were then replayed to the participants in the passive task, in which the participants only assignment was to listen to the sounds. For a better comparison between the ERPs from the active and the passive task, an additional motor control task was performed. Here, the participants were again asked to press the button at a steady rhythm; however, no sound was presented. The motor activity, as reflected in the motor control task ERP, was then subtracted from the active task ERP for comparability with the passive task ERP. A smaller unspecific auditory N1 (SanMiguel, Todd, et al. 2013) in the motor‐corrected active compared to the passive task was usually observed (Aliu et al. 2009; Bäß et al. 2008; Martikainen et al. 2005; Schafer and Marcus 1973). This effect was even stable when using feet instead of hands to press the button (van Elk et al. 2014) and proved to be dependent on the motor intention (Timm et al. 2014).

Another phenomenon obtained in the context of self‐ versus externally generated sounds is the elicitation of an omission N1 when a sound that is expected is omitted. In the classical omission N1 paradigm sounds are also produced by button presses. Manipulated is either the predictability of occurrence—a button press produces a sound in 80% or only in 50% of trials and no sound in the remaining trials (SanMiguel, Widmann, et al. 2013)—or the predictability of identity—a button press produces always the same, “single” sound or a different, “random” sound in 80% of the trials and again no sound in the remaining trials (Dercksen et al. 2020; SanMiguel, Saupe, et al. 2013). A negative deflection in the time range of the auditory N1 is observed in omission trials in the predictable conditions, the omission N1, which is interpreted as prediction error (and indirectly as evidence for predictive processing; note that a smaller omission N1 was also elicited in the random sound condition interpreted as unspecific prediction error). The interpretation of the omission N1 as a prediction error is supported by studies conducting simulations in the context of the predictive coding theory, which yielded sensory‐like signals to such omissions (Friston and Kiebel 2009).

As a result of the similarity in the overall study designs, in our previous study (Tast et al. 2024) we developed a combined paradigm to investigate if N1 suppression and omission N1 actually both reflect prediction‐related processes and if they are possibly related to the same underlying mechanisms. We combined N1 suppression and omission N1 paradigms by adding a passive replay condition to a predictability of occurrence omission N1 paradigm. We found N1 suppression of similar amplitude in both the predictable (80% sounds, 20% omissions) and unpredictable (50% sounds, 50% omissions) condition. Since the strength of the suppression in the active compared to the passive task did not differ, we concluded that the N1 suppression is not sensitive towards sound predictability. However, the occurrence of an omission N1 demonstrated that predictions on sound occurrence are represented during auditory sensory processing in the N1 time range. The occurrence in the predictable condition only showed that the omission N1 was modulated by sound predictability and prediction violation only in this condition and not in the unpredictable condition.

However, several studies reported evidence that the N1 suppression is sensitive to the predictability of sound identity. A study by Bäß et al. (2008) investigated changes in the N1 suppression related to the predictability of the tone onset and the tone frequency. They found the largest suppression effects when both onset and frequency were predictable and the weakest in the condition with a predictable onset but unpredictable frequency. The authors interpreted that even though the most important feature for overall suppression is the agency over producing the sounds, a small part of the forward model was also due to the expected identity of the sound. In another study Knolle et al. (2013) intermittently presented deviants (with a different frequency) instead of standards. They found a stronger suppression of the N1 in response to the standards than the deviants. These results support the interpretation that the N1 suppression is the result of a forward model that takes the identity of the expected stimulus into account. The apparent contradiction between our previous study and these results could be resolved if N1 suppression was not sensitive to predictability of occurrence but only sensitive to predictability of identity. On the other hand, several studies found no identity prediction related effects in the N1 suppression (Hughes et al. 2013; Lindner et al. 2024). Even though such results could also be explained by differences in the experimental design, it is important to investigate these contradictions further. Consequently, here we want to test this hypothesis by combining a predictability of identity N1 suppression and omission N1 paradigm. Additionally, using this paradigm will give us the opportunity to compare the omission N1 and the N1 suppression more closely.

In this study we aim to build upon the findings of previous research and directly compare the impact of the predictability of the identity of a sound on the N1 suppression and the omission N1. Therefore, in one condition the sound occurring in 80% of button presses was always the same, while in the other condition each sound was different. We analyzed the omission responses as well as the difference between the actively produced sounds and the passively perceived sounds. In addition to N1 related components we aimed to extend the analyses to ERP components succeeding the (omission) N1, to get a bigger picture of the influence of predictability on auditory processing for expected sounds and for unexpected omissions. Similar to omission N1 (oN1), also omission N2 (oN2, related to MMN by Dercksen et al. 2020) and omission P3 (oP3) components were reported for omissions of expected sounds (Dercksen et al. 2020; SanMiguel, Saupe, et al. 2013). In N1 suppression studies, also the P2 for predicted sounds was found to be suppressed (Horváth et al. 2012; Knolle et al. 2013). In contrast to the N1, which is regarded as reflecting sensorial processing, P2, oN2, and oP3 reflect temporarily later and presumably higher cognitive stages of processing expectancy violations.

Another interesting aspect we observed in our previous study was a left lateralization of the omission N1. Since the participants used their right hand only, we thought that this was a hand‐specific effect, in accordance with other preceding studies showing bilateral omission N1 for (within‐participant) balanced use of left and right hands (SanMiguel, Widmann, et al. 2013) versus left lateralization for use of the right hand only (Dercksen et al. 2020, 2022). Such a lateralization for sensory suppression effects in a functional magnetic resonance imaging (fMRI) study has been reported previously (Reznik et al. 2014). To investigate it formally, we decided to explicitly include left versus right hand as independent variables and have participants use both their left and their right hand in the experiment. If the omission N1 is indeed strongest contralateral to the hand that was used, then we expected to see a difference in topography for the left and the right hand.

Regarding the main research questions, we expected the omission N1 to be sensitive to the predictability of the sound identity. Thus, in the predictable condition, we expected to see a strong omission N1 and in the unpredictable condition no or at least a smaller omission N1 (similar to Dercksen et al. 2020). If the N1 suppression is sensitive to the predictability of the sound identity, the suppression should be stronger in the predictable condition (also in line with Bäß et al. 2008; Knolle et al. 2013). On the other hand, there is an increasing number of studies that challenge the hypothesis that N1 suppression for self‐generated sounds is based on a sound‐specific prediction (Egan et al. 2024; Han et al. 2022; Harrison et al. 2023; Horváth et al. 2012; Tast et al. 2024). Thus, similar N1 suppression in conditions with predictable vs. unpredictable sound identity could also be expected. This result would support our previous conclusion that both phenomena, N1 suppression and omission N1, presumably do not rely on a common (predictive) mechanism (Tast et al. 2024).

## 2 Methods

### 2.1 Participants

Thirty‐six participants (11 self‐identified as male and 25 as female, mean age = 25.4, age range: 18–33, 3 left‐handed) took part in the experiment. All reported normal hearing and normal or corrected to normal vision. The participants gave written informed consent for study participation, and they received a compensation of 8€/h or course credits for their participation. The Ethics Advisory Board of Leipzig University approved the study, and it was conducted in agreement with the Declaration of Helsinki.

### 2.2 Procedure

**FIGURE 1:**
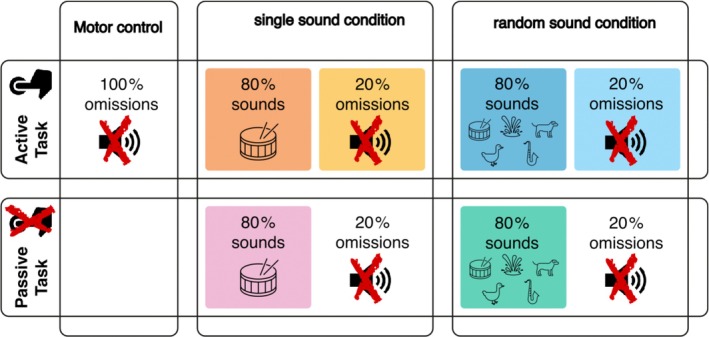
Experimental conditions and tasks. In an active task, participants pressed a button. Immediately after the button press, either the same tone was presented for the whole block (predictable single sound condition), or a new sound was presented after every button press (unpredictable random sound condition). In 20% of the button presses, no sound was presented (20% omission). In the passive task, the participants were asked to listen to a replay of the tones they produced in the active condition and did not have to perform any action. Additionally, there was a motor control condition where button presses did not produce any sounds.

The experimental design was similar to Tast et al. (2024). The experiment consisted of two main tasks: an active task, where the participants had to press a button with their index finger approximately every 2 s to produce a sound, and a passive task where the participants listened to a replay of the sounds they had produced in the active task. Participants used the left index finger for half of the blocks of each condition and the right index finger for the other half. The sounds were omitted in 20% of the button presses. Depending on the condition, the participants produced the same sound over the whole block (single sound condition) or a sound randomly selected from a set of 48 different sounds (random sound condition). This led to the sound identity being either predictable or unpredictable. A third condition without any tones being presented after the button presses served as the motor‐control condition. An overview of the experimental design can be seen in Figure 1. Both sound conditions contained 72 omissions and 288 sound trials, resulting in a total of 360 trials per condition. These were divided into 4 blocks with 90 trials (18 omissions, 72 sounds). The motor control condition was divided into two blocks with 72 trials each. The experiment always started with two training blocks containing 36 trials each. The first training block was without sounds and was done with the right hand, while in the second training block, the participants produced a sound when pressing the button with the left hand. The purpose of the training blocks was for the participants to get an understanding of the task and to learn the two‐second rhythm in that they had to press the button. During the two training blocks, the duration between button presses was always displayed on the screen, following each button press. Additionally, either “too fast” or “too slow” (in German language) was displayed when the duration was below 1.6 or longer than 2.4 s. A histogram containing the inter‐response intervals was displayed after each block.

The main experimental blocks always started and ended with one silent motor control block. Each two active sound blocks were always directly followed by the two corresponding passive replay blocks. The order of the conditions and hand used was counterbalanced between participants.

### 2.3 Stimuli and Apparatus

The sound stimuli in the experimental blocks consisted of 48 different common environmental sounds (e.g., a dog barking, a ship horn or a sneeze; rated as identifiable by an independent sample of participants, Wetzel et al. 2011) with a length of 200 ms including 10 ms rise and fall time (Dercksen et al. 2020, 2022; SanMiguel, Saupe, et al. 2013). Each single sound block used a different sound from the 48 potential sounds, and these were removed from the random sound condition for each participant individually. The training block used a simple sinusoidal sound with a length of 200 ms including a rise and fall time of 10 ms each. It had a frequency of 800 Hz and included the first two harmonics (1600 Hz, −3 dB and 2400 Hz, −6 dB). Tones were presented binaurally immediately after the button press via Sennheiser HD25 headphones at a loudness of 58 dB SPL.

A custom‐built infrared photoelectric button was used to avoid any additional acoustic stimulation. The participants used either their left or right index finger to press the button (depending on the block). The button was connected to the stimulus computer via a RTbox (Li et al. 2010). The participants were seated in a comfortable office chair inside a dimly lit, electrically shielded, double‐walled sound booth in front of a ViewPixx/EEG LCD computer screen (VPixx, QC, Canada). A black fixation cross (0.5° × 0.5° visual angle) was displayed at the center of the screen in front of a gray background. The feedback during the training blocks used black digits/letters and was presented above (time in seconds) and below (“too fast/too slow”) the fixation cross.

### 2.4 Data Recording

The electroencephalogram (EEG) was recorded using a 64 channel BrainAmp EEG amplifier and a 64 active electrode ActiCap (BrainProducts, Gilching, Germany). They were placed following a subset of the extended 10–10 system: FP1, FP2, AF7, AF3, AFz, AF4, AF8, F7, F5, F3, F1, Fz, F2, F4, F6, F8, FT7, FC5, FC3, FC1, FCz, FC2, FC4, FC6, FT8, T7, C5, C3, C1, Cz, C2, C4, C6, T8, TP7, CP5, CP3, CP1, CPz, CP2, CP4, CP6, TP8, P7, P5, P3, P1, Pz, P2, P4, P6, P8, PO7, PO3, POz, PO4, PO8, O1, O2 and the left and right mastoids (M1 and M2). A reference electrode was placed on the tip of the nose. Additionally, the horizontal electrooculogram (EOG) was recorded between the outer canthi of the two eyes (LO1, LO2) as well as the vertical EOG between FP1 and an infraorbitally placed electrode (IO1). The data was sampled at a rate of 500 Hz. The electrode impedances were kept below 20 kΩ. A high‐pass filter with a time constant of 10 s and a 250 Hz low‐pass filter were used for online filtering of EEG and EOG. Further the time for each button press was recorded.

### 2.5 Data Analysis

The data was preprocessed with EEGLAB (Delorme and Makeig 2004) using an offline 48 Hz low‐pass filter (403 point Kaiser‐windowed sinc FIR filter, Kaiser beta = 5, transition band width = 4 Hz) as well as a 0.1 Hz high‐pass filter (8025 point Kaiser windowed sinc FIR filter, Kaiser beta = 5, transition band width = 0.2 Hz; Widmann et al. 2015). For further analysis, the data was divided into epochs with a duration of 700 ms, starting 200 ms before the sound was delivered and ending 500 ms after sound onset. The first two trials after each omission were excluded as well as trials with a very short (< 1 s) or very long (> 3.5 s) sound onset asynchrony (SOA). Further, epochs where the signal exceeded 750 μV peak‐to‐peak amplitudes at any electrode were also excluded (to eliminate large non‐stereotypical artifacts such as swallowing but retain stereotypical artifacts such as blinking). Additionally, all channels with a robust z score of the robust standard deviation greater than 3 were rejected (Bigdely‐Shamlo et al. 2015). Channels FP1, FP2, M1, M2, LO1, LO2, and IO1 were never excluded. The number of included trials per task, condition, and participant can be found in Table 1.

**TABLE 1:** Number of included trials per task, experimental condition, and participant.

	Mean	SD	Min	Max
Motor control	68.9	2.2	59	70
Single sound active	68.5	2.2	61	72
Single sound passive	67.8	3	54	72
Single sound omission	35.5	1	30	36
Random sound active	68.9	1.9	61	72
Random sound passive	68.3	2.4	61	72
Random sound omission	35.6	0.7	33	36

In the next step, using the AMICA algorithm, an independent component analysis (ICA) was done to correct for ocular artifacts (Delorme et al. 2012). The preprocessing for this data was only changed in one way: a 1 Hz high‐pass filter (1605 point Kaiser‐windowed sinc FIR filter, Kaiser beta = 5, transition band width = 1 Hz) was used instead of the 0.1 Hz high‐pass filter. Ocular artifact ICs, specifically blink‐related artifacts and eye movements (pre‐saccadic spike potentials and horizontal and vertical movements of the corneo‐retinal dipoles; Plöchl et al. 2012) were pre‐classified using the ICLabel EEGLAB plugin (Pion‐Tonachini et al. 2019) and selected manually. Three to five blink‐and eye‐movement‐related ICs per participant were removed from the data (mean = 4.33, median = 4). Between zero and four additional muscle activity‐related or channel noise ICs causing many epoch rejections were manually selected and removed per participant (mean = 0.2). The manual selection of all artifact ICs was guided by the premise of avoiding as much as possible the rejection of components that showed neural contributions (e.g., an alpha peak in the spectrum or an ERP). In total, an average of 4.53 ICs per participant were removed from the data set (median = 4, min = 3, max = 9).

**FIGURE 2:**
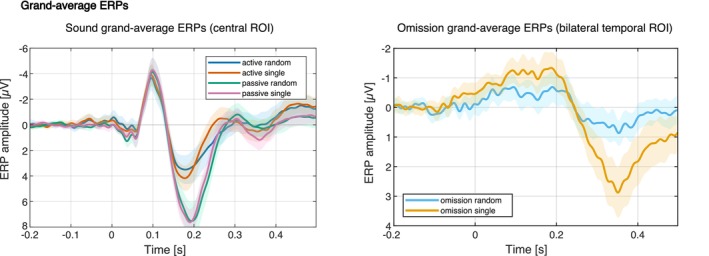
Grand average ERPs for the sound trials (central ROI, left) and omission trials (bilateral temporal ROI, right). Shaded areas reflect 95% confidence intervals.

In the next step, the artifact IC activity was subtracted from the 0.1 Hz high‐pass filtered data, and then the data was baseline corrected for the time window of −0.2 s to −0.1 s. All epochs with peak‐to‐peak amplitudes larger than 150 μV were excluded before individual average ERPs were computed for all conditions (single sound and omission trials, random sound and omission trials, motor control as well as the corresponding passive replay for the conditions) for every participant. Using the individual ERPs, grand average waveforms per stimulus type were computed for visualization. They are depicted in Figure 2.

### 2.6 Statistical Analysis

**FIGURE 3:**
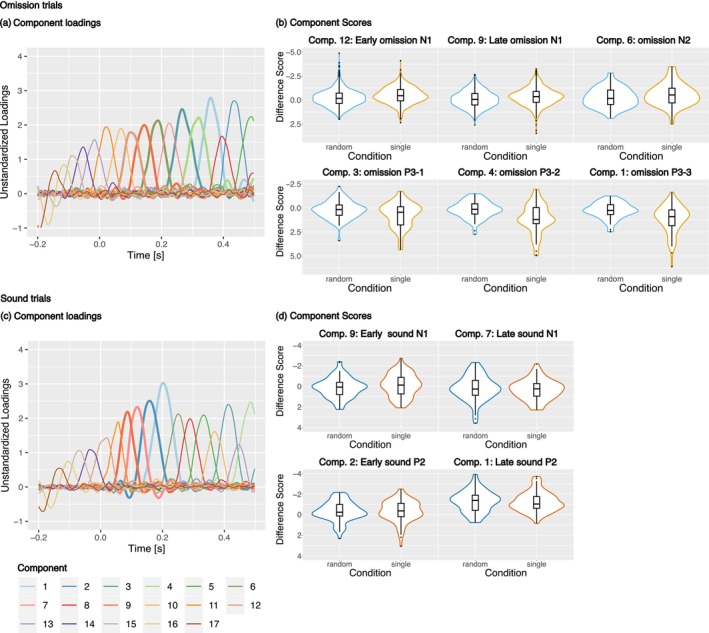
PCA component loadings (a, c) and violin and box plots (b, d) for the omission (top) and sound (bottom) trials. Omission PCA components 1, 3, 4, 6, 9, and 12 were associated with the omission P3‐3, omission P3‐1, omission P3‐2, omission N2, late omission N1, and early omission N1 respectively. The sound components 1, 2, 7, and 9 were associated with the late and early P2 and the late and early N1, respectively. The loadings of the components selected for both analyses are indicated by a thicker line. The component scores reflected in the violin plots were corrected by subtracting the scores of the motor‐control condition per condition (i.e., omission minus motor‐control and [active minus motor‐control] minus passive, respectively).

To isolate the sound and omission components we used temporal PCA (R psych package, Revelle 2023). PCA has the advantage of isolating ERP components from potentially overlapping components (e.g., shifting peak latencies) as well as allowing a data‐driven approach to the derivation of the dependent variables, reducing potential user biases for example in the selection of time windows. Two separate PCAs for the sound trials (plus motor‐control) and omission trials (plus motor‐control) were performed with the help of the tutorial code by Scharf and colleagues (Scharf et al. 2022). To determine the number of components we used Horn's parallel test (17 components for both PCAs). Figure 3 displays the component loadings and component scores for both the sound and omission trial PCAs. In the next step the data was rotated with the Geomin rotation. The mean component scores for the sound N1 were computed in a central region of interest (ROI; FC1, FCz, FC2, C1, Cz, C2, CP1, CPz, CP2) while the mean component scores for the omission N1 were computed in two bilateral temporal ROIs (FT7, T7, TP7, FC5, C5, CP5 and FT8, T8, TP8, FC6, C6, CP6). A frontocentral ROI (FC1, FCz, FC2, C1, Cz, C2) was used to compute the mean component scores for the sound P2 and the omission P3‐1, as well as a frontal ROI (F3, F4, Fz, FC1, FC2) for the oN2 and a centro‐parietal region (Cz, CP1, CP2, Pz) for the omission P3‐2 and the omission P3‐3. The ROIs were selected based on the spatial peaks in the topographies of the different components. They are mostly in line with the literature (Dercksen et al. 2020; Lange 2009; Timm et al. 2014). We expected to find a sound evoked N1 with a peak at vertex electrode locations around 100 ms after stimulus onset (like Tast et al. 2024) and a sound P2 similar to Horváth (2013). Additionally, we expected omission component structures similar to Dercksen et al. (2020).

The design of the analyses of the omission components is not balanced. As in previous studies (Dercksen et al. 2020; Tast et al. 2024), to test whether an omission N1 was elicited in the single and random sound conditions, we first compared the component scores of the omission trials in the active task to the motor‐control task separately for both the single and the random sound conditions in two 2 × 2 × 2 ANOVAs with the factors condition (motor vs. single and motor vs. random, respectively), hemisphere (left vs. right), and hand (left vs. right). In a second step, to test whether the omission N1 amplitudes differed between single and random sound conditions, we directly compared the (motor‐corrected) omission trials of the single sound condition to the (motor‐corrected) trials of the random sound condition in a 2 × 2 × 2 ANOVA with the factors condition (random vs. single), hemisphere (left vs. right), and hand (left vs. right).

To compare the sound responses of the active to those from the passive task, the motor‐control condition was subtracted from the active task components to correct for motor activity. Afterwards, the component scores of the active sound responses were compared directly to the passive sound component scores in a 2 × 2 × 2 ANOVA with the factors condition (random vs. single), task (active vs. passive), and hand (left vs. right).

Using the BayesFactor package in R (Morey and Rouder 2022), Bayesian ANOVAs were computed for all omission and sound comparisons. As part of the factorial design, the ANOVAs included all factors as main effects as well as their interactions (including three‐way interactions).

A BF_10_ larger than 3 (or lower than 0.33) was interpreted as moderate evidence in favor of the alternative (null) hypothesis, and a BF_10_ larger than 10 (or lower than 0.1) was interpreted as strong evidence in favor of the alternative (null) hypothesis (Lee and Wagenmakers 2014).

## 3 Results

### 3.1 Behavior

On average participants performed well keeping the 2 s rhythm and pressed the button every 2.013 s (SD = 0.2764 s, min = 0.159 s, max = 5.719 s). The participants were on average slightly slower in the motor control condition (2.087 s, SD = 0.342 s) but very precise in both the single sound (2.013 s, SD = 0.267 s) and the random sound (1.999 s, SD = 0.269 s) condition.

### 3.2 Omission N1

**FIGURE 4:**
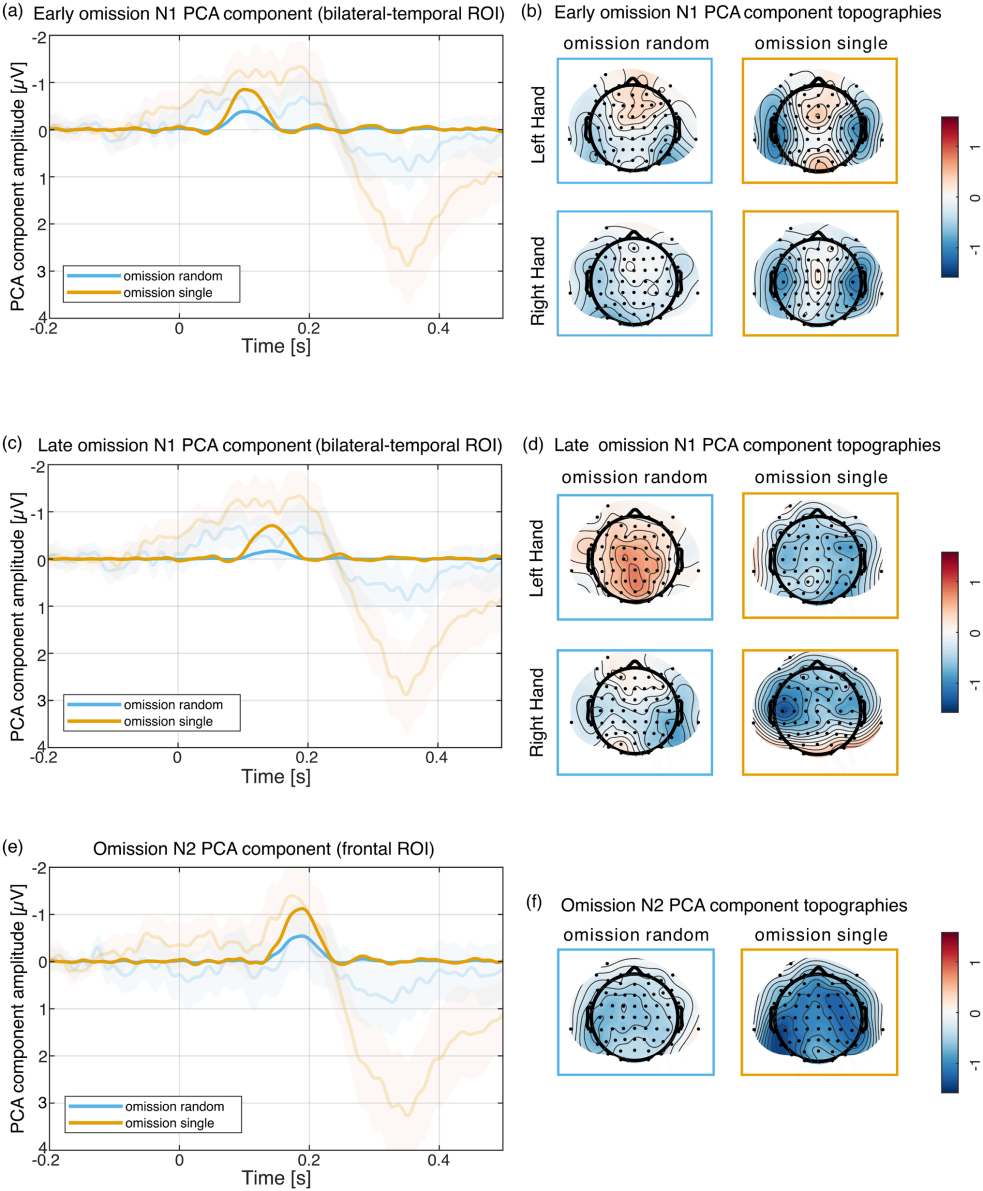
Omission (minus motor‐control) PCA component time courses (unstandardized component loadings multiplied by component scores) and topographies (unstandardized component loading peak amplitudes multiplied by component scores) for the early omission N1 (a, b), the late omission N1 (c, d) and the omission N2 (e, f). In the random sound condition an omission component was observed for the early omission N1 and the omission N2 only, but in the single sound condition an omission component was observed for the early omission N1, the late omission N1 and the omission N2.

The PCA separated the omission N1 into two components (as previously reported by Dercksen et al. 2022; Korka et al. 2020). Component 12 reflecting the early omission N1 peaked at 100 ms and component 9 reflecting the late omission N1 peaked at 144 ms. Both components showed highest amplitudes at bilateral temporal electrode locations as can be seen in the respective topographies in Figure 4.

For the early omission N1 component the Bayesian ANOVAs comparing random sound and single sound conditions vs. motor‐control condition both preferred the model including the condition, hemisphere, and hand main effects and the hemisphere by hand interaction effect (random vs. motor: BF_10_ = 8 × 10^4^; single vs. motor: BF_10_ = 5.8 × 10^13^). The data provided strong evidence for an omission N1 in the random sound condition (random vs. motor: 0.03 vs. 0.42 μV, BF_incl_ = 12.7) and an omission N1 in the single sound condition (single vs. motor: −0.43 vs. 0.42 μV, BF_incl_ = 9.2 × 10^9^). The data showed more positive amplitudes over the right hemisphere compared to the left hemisphere for left hand responses but similar amplitudes between the hemispheres for right hand responses (random vs. motor: hemisphere BF_incl_ = 133, hand BF_incl_ = 5.06, hemisphere by hand BF_incl_ = 17.4; single versus motor: hemisphere BF_incl_ = 27, hand BF_incl_ = 12.5, hemisphere by hand BF_incl_ = 430). Importantly, against our hypothesis, the data did provide moderate evidence against a modulation of the omission N1 topography by hand and hemisphere (all 2‐ and 3‐way interactions including condition by hand or hemisphere BF_incl_ < 0.265).

For the early omission N1 component the Bayesian ANOVA comparing random sound versus single sound conditions preferred the model including the condition main effect only (random vs. single: −0.39 vs. −0.85 μV, BF_10_ = 17.3). The data provided strong evidence for a larger omission N1 amplitude in the single sound condition compared to the random sound condition (BF_incl_ = 17.3) and moderate evidence against all other main and interaction effects (all BF_incl_ < 0.262).

For the late omission N1 component, the Bayesian ANOVA comparing random sound versus motor‐control condition preferred the null model. The data provided moderate evidence against an omission N1 in the random sound condition (random vs. motor: 0.09 vs. 0.26 μV, BF_incl_ = 0.264). The Bayesian ANOVA comparing single sound versus motor‐control condition preferred the model including the condition and hemisphere main effects (BF_10_ = 2.7 × 10^4^). The data provided strong evidence for an omission N1 in the single sound condition (single vs. motor: −0.45 vs. 0.26 μV, BF_incl_ = 2.4 × 10^4^) and anecdotal evidence for more positive ERP amplitudes over the right hemisphere compared to the left hemisphere ROI (left vs. right hemisphere: 0.05 vs. −0.25 μV; BF_incl_ = 1.33) and anecdotal to moderate evidence against all other main and interaction effects (all BF_incl_ < 0.425).

For the late omission N1 component, the Bayesian ANOVA comparing random sound versus single sound conditions preferred the model including the condition and hand main effects (BF_10_ = 48.1). The data provided strong evidence for a larger omission N1 amplitude in the single sound condition compared to the random sound condition (random vs. single: −0.17 vs. −0.71 μV, BF_incl_ = 18), anecdotal evidence for more negative ERPs for right hand compared to left hand responses (left vs. right hand: −0.22 vs. −0.65 μV, BF_incl_ = 2.9), and anecdotal to moderate evidence against all other main and interaction effects (all BF_incl_ < 0.379).

### 3.3 Omission N2

Component 6 of the omission PCA reflected the omission N2. It peaked at 188 ms over fronto‐central electrodes and is shown in Figure 4.

For the omission N2 component, the Bayesian ANOVAs comparing random sound and single sound conditions versus motor‐control condition both preferred the model including the condition main effect only (random vs. motor: BF_10_ = 1.78; single vs. motor: BF_10_ = 345). The data provided anecdotal evidence for an omission N2 in the random sound condition (random vs. motor: 0.69 vs. 1.23 μV, BF_incl_ = 1.78), strong evidence for an omission N2 in the single sound condition (single vs. motor: 0.11 vs. 1.23 μV, BF_incl_ = 343), and anecdotal to moderate evidence against all other main and interaction effects (all BF_incl_ < 0.397).

For the omission N2 component the Bayesian ANOVA comparing random sound versus single sound conditions preferred the null model. The data did not provide conclusive evidence for a larger omission N2 amplitude in the single sound condition compared to the random sound condition (random vs. single: −0.54 vs. −1.12 μV, BF_incl_ = 0.705), and moderate evidence against all other main and interaction effects (all BF_incl_ < 0.244).

### 3.4 Omission P3

**FIGURE 5:**
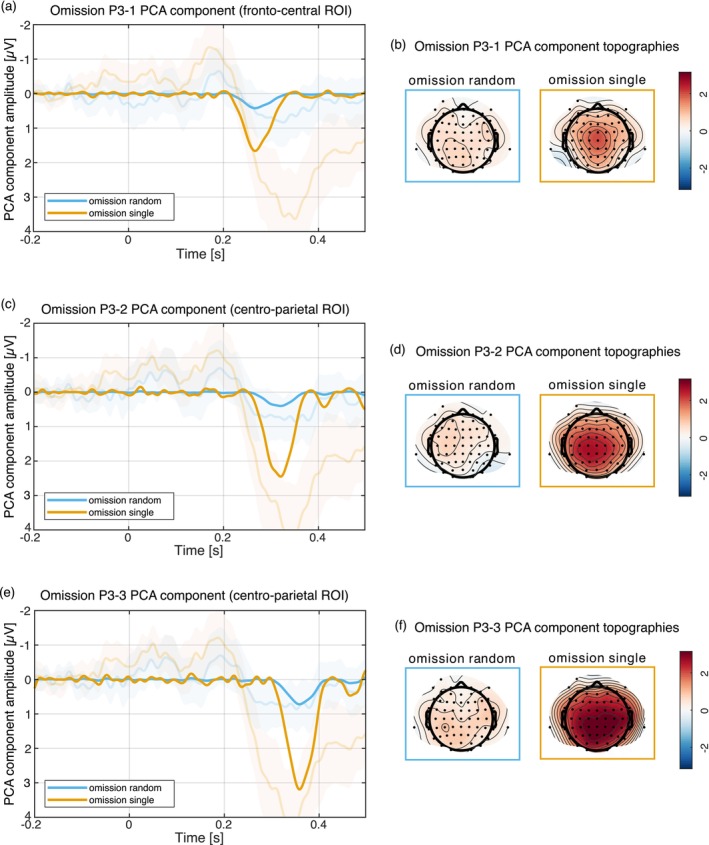
Omission PCA component time courses and topographies for the three subcomponents of the omission P3, the omission P3‐1 (a, b), the omission P3‐2 (c, d) and omission P3‐3 (e, f). For the random sound condition, only an omission P3‐3 was observed, but the single sound condition elicited an omission P3‐1, P3‐2, and P3‐3.

The omission P3 was separated into three subcomponents by the PCA, the oP3‐1, oP3‐2, and oP3‐3 reflected by components 3, 4, and 1 respectively (see Dercksen et al. 2020, 2022). Component 3 peaked at 266 ms and was strongest over central electrodes. Component 4 peaked at 320 ms over centro‐parietal electrodes. Component 1 peaked at 358 ms also over centro‐parietal electrodes. This can be seen in Figure 5.

For the omission P3‐1 component, the Bayesian ANOVA comparing random sound versus motor‐control condition preferred the null model. The data did not provide conclusive evidence for an omission P3‐1 in the random sound condition (random vs. motor: 2.11 vs. 1.61 μV, BF_incl_ = 0.889). The Bayesian ANOVA comparing single sound versus motor‐control condition preferred the model including the condition main effect only (BF_10_ = 1.6 × 10^4^). The data provided strong evidence for an omission P3‐1 in the single sound condition (single vs. motor: 3.44 vs. 1.61 μV, BF_incl_ = 1.6 × 10^4^) and anecdotal to moderate evidence against all other main and interaction effects (all BF_incl_ < 0.473).

For the omission P3‐1 component the Bayesian ANOVA comparing random sound versus single sound conditions preferred the model including the condition main effect only (BF_10_ = 22.4). The data provided strong evidence for a larger omission P3‐1 amplitude in the single sound condition compared to the random sound condition (random vs. single: 0.5 vs. 1.82 μV, BF_incl_ = 22.5) and anecdotal to moderate evidence against all other main and interaction effects (all BF_incl_ < 0.5).

For the omission P3‐2 component, the Bayesian ANOVA comparing random sound versus motor‐control condition preferred the model including the hand main effect only (BF_10_ = 2.44). The data did not provide conclusive evidence for an omission P3‐2 in the random sound condition (random vs. motor: 1.81 vs. 1.41 μV, BF_incl_ = 0.675) but provided anecdotal evidence for more positive ERP amplitudes following left hand responses compared to right hand responses (left vs. right hand: 2.56 vs. 2.16 μV, BF_incl_ = 2.51). The Bayesian ANOVA comparing single sound versus motor‐control condition preferred the model including the condition main effect only (BF_10_ = 7 × 10^7^). The data provided strong evidence for an omission P3‐2 in the single sound condition (single vs. motor: 3.86 vs. 1.41 μV, BF_incl_ = 7.1 × 10^7^) and anecdotal to moderate evidence against all other main and interaction effects (all BF_incl_ < 0.453).

For the omission P3‐2 component, the Bayesian ANOVA comparing random sound vs. single sound conditions preferred the model including the condition main effect only (BF_10_ = 1.8 × 10^4^). The data provided strong evidence for a larger omission P3‐2 amplitude in the single sound condition compared to the random sound condition (random vs. single: 0.4 vs. 2.45 μV, BF_incl_ = 1.8 × 10^4^) and anecdotal to moderate evidence against all other main and interaction effects (all BF_incl_ < 0.381).

For the omission P3‐3 component, the Bayesian ANOVA comparing random sound and single sound conditions vs. motor‐control condition both preferred the model including the condition main effect only (random vs. motor: BF_10_ = 3.27; single vs. motor: BF_10_ = 8.5 × 10^7^). The data provided moderate evidence for an omission P3‐3 in the random sound condition (random vs. motor: 2.19 vs. 1.47 μV, BF_incl_ = 3.39), strong evidence for an omission P3‐3 in the single sound condition (single vs. motor: 4.66 vs. 1.47 μV, BF_incl_ = 8.7 × 10^7^), and anecdotal to moderate evidence against all other main and interaction effects (all BF_incl_ < 0.872).

For the omission P3‐3 component, the Bayesian ANOVA comparing random sound versus single sound conditions preferred the model including the condition main effect only (BF_10_ = 2.7 × 10^4^). The data provided strong evidence for a larger omission P3‐3 amplitude in the single sound condition compared to the random sound condition (random vs. single: 0.72 vs. 3.19 μV, BF_incl_ = 2.7 × 10^4^) and anecdotal to moderate evidence against all other main and interaction effects (all BF_incl_ < 0.821).

### 3.5 N1 Suppression

**FIGURE 6:**
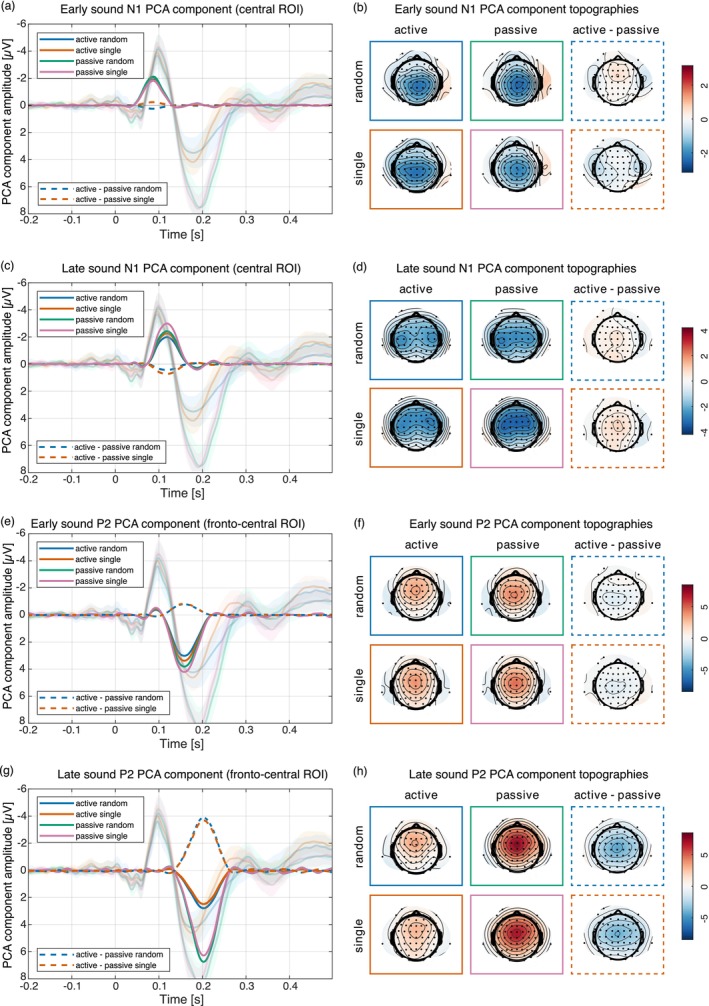
Sound PCA component time courses and topographies for the early (a, b) and late (c, d) N1 and early (e, f) and late (g, h) P2. The active condition PCA components were motor corrected. For the N1 components no suppression of the active compared to the passive components can be seen, however for the P2 components such a suppression is clearly visible. This is also represented by the dashed lines. The strength of suppression was not modulated by the predictability of the sound identity.

The PCA separated the sound N1 into two subcomponents, component 9 with a peak at 86 ms and component 7 with a peak at 118 ms. Figure 6 shows that both components display the highest amplitudes at central electrode locations.

For the early N1 component the Bayesian ANOVA preferred the null model. The data provided moderate evidence against a main effect of sound N1 suppression in the active compared to the passive task (active vs. passive: −1.96 vs. −1.97 μV, BF_incl_ = 0.128) and against a main effect of sound type (random vs. single: −2 vs. −1.93 μV, BF_incl_ = 0.136) and anecdotal evidence against an interaction effect of task and sound type (BF_incl_ = 0.373; note that numerically in the single sound condition we observed an N1 enhancement rather than N1 suppression in the active compared to the passive task). The data provided moderate evidence against any main or interaction effect including hand (all BF_incl_ < 0.257).

For the late N1 component, the Bayesian ANOVA preferred the model including the task main effect only (BF_10_ = 4.08). The data provided moderate evidence for a main effect of sound N1 suppression in the active compared to the passive task (active vs. passive: −2.12 vs. −2.71 μV, BF_incl_ = 4.19), inconclusive evidence against a main effect of sound type (random vs. single: −2.21 vs. −2.63 μV, BF_incl_ = 0.829), and, importantly, moderate evidence against an interaction effect of task and sound type (BF_incl_ = 0.248). The data provided moderate evidence against any main or interaction effect including hand (all BF_incl_ < 0.226).

### 3.6 P2 Suppression

The PCA also separated the sound P2 into two components, component 2 with a peak at 158 ms and component 1 with a peak at 202 ms. Both components showed highest amplitudes at fronto‐central electrode locations as can be seen in Figure 6.

For the early P2 component the Bayesian ANOVA preferred the model including the task main effect only (BF_10_ = 163). The data provided strong evidence for a main effect of sound P2 suppression in the active compared to the passive task (active vs. passive: 3.12 vs. 3.93 μV; BF_incl_ = 172), inconclusive evidence against a main effect of sound type (random vs. single: 3.32 vs. 3.74 μV, BF_incl_ = 0.889), and, importantly, moderate evidence against an interaction effect of task and sound type (BF_incl_ = 0.176). The data provided moderate evidence against any main or interaction effect including hand (all BF_incl_ < 0.26).

For the late P2 component the Bayesian ANOVA also preferred the model including the task main effect only (BF_10_ = 1.4 × 10^29^). The data provided strong evidence for a main effect of sound P2 suppression in the active compared to the passive task (active vs. passive: 2.55 vs. 6.38 μV; BF_incl_ = 1.5 × 10^29^) and moderate evidence against a main effect of sound type (random vs. single: 4.63 vs. 4.3 μV, BF_incl_ = 0.26), and, importantly, against an interaction effect of task and sound type (BF_incl_ = 0.192). The data provided moderate evidence against any main or interaction effect including hand (all BF_incl_ < 0.256).

## 4 Discussion

In this study we wanted to investigate whether N1 suppression is sensitive to the predictability of sound identity and thus, whether N1 suppression and omission N1 may possibly be based on the same mechanism. In an active task participants repeatedly pressed a button. In 80% of trials the button press elicited a sound, in 20% of trials the sound was unexpectedly omitted. The identity of the sound was either predictable (the same sound for all trials within a block) or unpredictable (a different sound for each trial within a block) in separate blocked conditions. We found a larger omission N1 to sound omissions in the predictable condition compared to the unpredictable condition. We also found reduced N1 and P2 amplitudes in response to sounds in the active compared to the passive task, that is N1 and P2 suppression. However, the level of suppression was not modulated by the predictability of sound identity for either N1 or P2 amplitudes.

### 4.1 Omission Components Are Sensitive to Predictability of Sound Identity

We identified six omission components: an early and a late omission N1, an omission N2, and three omission P3 components, the oP3‐1, oP3‐2, and oP3‐3 (the nomenclature used by Dercksen et al. 2020, 2022; SanMiguel, Widmann, et al. 2013 indicates the time range relative to established ERP components and polarity of the omission responses). All components showed a sensitivity towards the predictability of sound identity reflected by a larger component in the single sound condition and a smaller or absent component in the random sound condition. This replicates earlier findings (Dercksen et al. 2020, 2022; SanMiguel, Saupe, et al. 2013). The larger omission component in the single sound condition can be interpreted as a violation of a more specific prediction compared to the random sound condition, in which only nonspecific information about sound occurrence, but not about sound identity, was available. Therefore, not only the information regarding *whether* a sound will occur but also the information regarding *which* sound will occur is relevant for the predictive mechanism underlying omission N1.

The omission N2 can be interpreted as an indicator for error evaluation and deviance detection (SanMiguel, Saupe, et al. 2013; van Laarhoven et al. 2017). Dercksen et al. (2020, 2024) suggested a potential relation of omission N2 to the frontal aspect of the mismatch negativity (MMN; Garrido et al. 2009; Näätänen et al. 2007; Schröger et al. 2014). An omission N2 in both conditions indicates that an error or a deviation from the expected input was perceived, however it was stronger in the single sound condition. The omission P3 was previously associated with attentional orienting and model updating to minimize prediction error in the future (Dercksen et al. 2020, 2022, 2024; SanMiguel, Saupe, et al. 2013; van Laarhoven et al. 2017). The single sound condition elicited an omission P3 in all three subcomponents, while the random sound condition only elicited an omission P3‐3. This could be interpreted as model updating happening for both conditions, but on a much larger scale in the single sound condition.

### 4.2 Sensory Suppression Is Not Sensitive to Predictability of Sound Identity

The PCA separated both the sound N1 and the sound P2 into an early and a late (sub‐) component. For the N1 three different subcomponents of the N1, the Na, the T‐complex (with spatial peaks over temporal sites) and the unspecific N1 have been proposed (Näätänen and Picton 1987; SanMiguel, Todd, et al. 2013). When comparing our topographies with those reported by SanMiguel, Todd, et al. (2013), who investigated which parts of the N1 were affected by sensory suppression, it can be assumed that the early component presumably reflects the Na, while the late N1 represents the unspecific N1.

The early N1 showed no signs of sensory suppression in the active compared to the passive task, while the late N1 and both the early and late P2 were smaller in the active task than in the passive task. Crucially, the strength of suppression was not influenced by the predictability of sound identity for any of the components, and the data rather provide evidence for similar suppression amplitudes in both conditions.

The two different interpretations for the cause of N1 suppression—reduced prediction error due to specific predictions versus sensory gating—were discussed in detail by Schröger et al. (2015). They concluded that there appears to be evidence for both interpretations. In our studies, we could not find any effects of the predictability of sound occurrence (Tast et al. 2024) or sound identity (present study). While the occurrence of suppression in both the N1 and the P2 replicates previous studies (Baess et al. 2011; Horváth et al. 2012; Knolle et al. 2019; SanMiguel, Todd, et al. 2013; Tast et al. 2024), the absence of an effect of predictability contradicts the results reported by Knolle et al. (2013) and Bäß et al. (2008). It should be noted that the evidence for an effect of predictability on N1 suppression provided by the data reported by Bäß et al. (2008; BF_10_ = 2, computed from the reported F‐value for the “frequency” [suppression for predictable vs. unpredictable] main effect with R BayesFactor package function meta.ttestBF and the default “medium” effect size prior; Morey and Rouder, 2022) and Knolle et al. (2013; BF_10_ = 1.24, computed from the reported F‐value for the “DIFF‐TYPE” [suppression for standard vs. deviant sound types] main effect) is limited (anecdotal or inconclusive).

There are two evident disparities between our study and the ones by Knolle et al. (2013) and Bäß et al. (2008) that could explain the contradictory results. The most obvious is that the present study included omissions, while the two previous studies did not. The occurrence of omission ERPs in fact demonstrates that a sound is still expected after every button press. Another difference is that we use wideband environmental sounds while they both used narrowband sine wave sounds. However, most studies that did not find prediction related suppression effects also used sine wave sounds (e.g., Han et al. 2022; Hughes et al. 2013; Lindner et al. 2024; Tast et al. 2024). It is therefore unlikely that the differences in the experimental design can explain the disparity in the results and it is important to discuss alternative explanations.

This view is supported by other studies not finding effects of predictability on N1 suppression and no or only small suppression effects in the P2 related to identity (Hughes et al. 2013; Lindner et al. 2024) or action‐effect contingency (Han et al. 2022; Harrison et al. 2023; Egan et al. 2024). In the study by Lindner et al. (2024) they presented tones that were congruent and incongruent to action‐effects learned and tested in subsequent phases. They found small suppression effects for the incongruent compared to the congruent condition on the P2 and no effects on the N1. The authors concluded that sensory suppression is not always present in forward modeling and further that identity‐specific action‐effect prediction might not be represented in the N1. In a similar study, Hughes et al. (2013) did not observe N1 differences between predictable and unpredictable sounds. They proposed the idea that effects of predictability might be related to the level of uncertainty, and they possibly did not find identity prediction effects because the uncertainty was very small. This explanation however seems less likely in the light of the present study where 44 different sounds would have led to a high uncertainty and yet we also found no effects of identity prediction on N1 suppression. It is important to note that while Lindner et al. (2024) and Hughes et al. (2013) did not find N1 suppression effects related to predictability of identity, Hughes et al. (2013) did report effects of prediction‐congruency on N1. In a recent study by Berryman et al. (2025) demonstrating N1 suppression for inner speech‐like self‐generated “sounds” again no effects of predicted sound intensity were found. Studies by Han et al. (2022) and Harrison et al. (2023) did not yield a modulation of the N1 amplitude by action‐effect contingency. Both studies had a predictable condition where the decision to press a button always produced a sound and further an unpredictable condition with a 50% sound occurrence probability after a button press. The N1 amplitudes between the predictable and the unpredictable condition did not differ significantly. Egan et al. (2024) conducted a similar study where they had participants either press a button (active condition) or watch a picture of a button press (observed active condition) which resulted in a sound in 50% of the cases. Additionally, in a passive condition with the same sound occurrence likelihood the sound was either cued or uncued. The authors found a suppressed N1 for the active task and the observed active task, and the difference between these two conditions was not significant. Interestingly the cued and the uncued passive task N1 amplitudes also did not differ significantly to each other, which corroborates the theory that N1 suppression is a motor‐effect and contradicts the hypothesis that it is related to sound predictability.

Taken together, the prevailing hypothesis posits that the N1 suppression is the result of action‐effect coupling, where self‐produced stimuli are more expected and therefore less processed (Aliu et al. 2009; Baess et al. 2011; Schafer and Marcus 1973). However, the key question is whether the nature of the suppression effect is unspecific or specific with respect to the characteristics (identity, probability of occurrence) of the expected sound action effect. On the one hand, the hypothesis that N1 suppression reflects a specific motor prediction mechanism is supported by several studies: N1 suppression is sensitive to manipulation of the predictability of sound identity and sound onset (Bäß et al. 2008; Knolle et al. 2013). Further, mice showed a suppression effect only for sounds that were associated with a specific action but not for novel sounds (Audette and Schneider 2023). Timm et al. (2014) reported that N1 suppression only occurred for voluntary button presses, and thus motor planning is a necessary (or possibly sufficient; cf. Berryman et al. 2025) condition. This could be taken as an additional index of the representation of specific predictions. On the other hand, a growing body of evidence highlights that N1 suppression is unlikely to be prediction related; instead, it might reflect unspecific motor suppression of auditory sensory processing. These studies found no sensitivity in the strength of suppression towards different manipulations of predictability and are corroborated by studies showing that neither attention (Timm et al. 2013) nor sense of agency (Timm et al. 2016) has an effect on the strength of N1 suppression, and N1 suppression also occurs if motor response and sound only coincide and are not causally related (Horváth et al. 2012).

Although the exact nature of the N1 suppression remains unclear, it seems that prediction plays a smaller role than previously assumed. However, in a more general sense, also unspecific motor suppression or sensory gating as the most plausible hypothesis for N1 suppression is presumably predictive in nature. Own actions are potentially related to sensory input; therefore, it is a reasonable strategy to suppress sensory processing following the planning and execution of own actions in order to better discriminate self‐produced from external sensations and prioritize resources accordingly. Importantly, the underlying mechanism apparently is insensitive to any specifications of the action‐effect contingencies. These contingencies are undoubtedly represented in the auditory system in the N1 time range, as demonstrated by the omission N1, which was found to be not only sensitive towards the predictability of the occurrence of a self‐generated sound (Tast et al. 2024) but also towards the predictability of the identity of the expected sound (present study) and even by other studies on sensory suppression, for example, Audette and Schneider (2023). Therefore, N1 suppression and omission N1 are presumably based on different mechanisms; one that is related to sensory predictions (respectively their violation) of different specificity and one that is most likely a result of action‐related unspecific sensory gating. The reason why some studies (Bäß et al. 2008; Knolle et al. 2013) but not others (Egan et al. 2024; Han et al. 2022; Harrison et al. 2023; Hughes et al. 2013; Lindner et al. 2024) reported N1 suppression to be modulated by predictability, for example, possibly related to adaptation and/or deviance processing, remains to be resolved.

### 4.3 Omission N1 Is Not Lateralized (Contralateral to the Acting Hand)

Earlier studies found a left‐lateralization of the omission N1 whenever participants only used their right hand for the study (Dercksen et al. 2020, 2022). To investigate whether this lateralization is indeed hand‐specific, we asked participants in this study to alternate hands between blocks. Interestingly, we found no evidence for any kind of lateralization of the omission N1 in dependence on the used hand in the data. The left‐lateralization that was observed in Tast et al. (2024) was not replicated in the present study. Thus, the underlying cause for the apparent lateralization in some but not in other studies is also still to be resolved.

## 5 Conclusion

In summary, we have shown that all omission components were sensitive to the predictability of sound identity and with enhanced prediction error responses for the more predictable single sound condition. This indicates that action‐related predictive information is represented in the auditory system in the time range of the N1. The sound‐evoked components, on the other hand, did not hint at a sensitivity to the predictability of sound identity. Although both the N1 and the P2 were found to be suppressed in active versus passive tasks, the amount of suppression did not differ in strength between the sound predictability conditions. Therefore, we conclude that omission responses presumably reflecting (specific) prediction error and suppression effects presumably reflecting (unspecific) sensory gating are based on different mechanisms. The mechanisms underlying omission responses include sensory predictions about sound identity and occurrence, whereas those underlying the suppression of self‐produced sounds do not.

## Author Contributions

**Valentina Tast:** conceptualization, methodology, data curation, formal analysis, investigation, writing – original draft, writing – review and editing, visualization. **Erich Schröger:** conceptualization, methodology, supervision, writing – review and editing. **Andreas Widmann:** conceptualization, formal analysis, methodology, supervision, visualization, writing – review and editing.

## Conflicts of Interest

The authors declare no conflicts of interest.

## Data Availability

The data that support the findings of this study are available from the corresponding author upon request.
